# A comprehensive system for evaluation of remote sequence similarity detection

**DOI:** 10.1186/1471-2105-8-314

**Published:** 2007-08-28

**Authors:** Yuan Qi, Ruslan I Sadreyev, Yong Wang, Bong-Hyun Kim, Nick V Grishin

**Affiliations:** 1 Howard Hughes Medical Institute, University of Texas Southwestern Medical Center, 5323, Harry Hines Blvd, Dallas, TX 75390-9050, USA; 2 Department of Biochemistry, University of Texas Southwestern Medical Center, 5323, Harry Hines Blvd, Dallas, TX 75390-9050, USA; 3 Lineberger Comprehensive Cancer Center, University of North Carolina at Chapel Hill, Chapel Hill, NC 27599, USA

## Abstract

**Background:**

Accurate and sensitive performance evaluation is crucial for both effective development of better structure prediction methods based on sequence similarity, and for the comparative analysis of existing methods. Up to date, there has been no satisfactory comprehensive evaluation method that (i) is based on a large and statistically unbiased set of proteins with clearly defined relationships; and (ii) covers all performance aspects of sequence-based structure predictors, such as sensitivity and specificity, alignment accuracy and coverage, and structure template quality.

**Results:**

With the aim of designing such a method, we (i) select a statistically balanced set of divergent protein domains from SCOP, and define similarity relationships for the majority of these domains by complementing the best of information available in SCOP with a rigorous SVM-based algorithm; and (ii) develop protocols for the assessment of similarity detection and alignment quality from several complementary perspectives. The evaluation of similarity detection is based on ROC-like curves and includes several complementary approaches to the definition of true/false positives. Reference-dependent approaches use the 'gold standard' of pre-defined domain relationships and structure-based alignments. Reference-independent approaches assess the quality of structural match predicted by the sequence alignment, with respect to the whole domain length (global mode) or to the aligned region only (local mode). Similarly, the evaluation of alignment quality includes several reference-dependent and -independent measures, in global and local modes. As an illustration, we use our benchmark to compare the performance of several methods for the detection of remote sequence similarities, and show that different aspects of evaluation reveal different properties of the evaluated methods, highlighting their advantages, weaknesses, and potential for further development.

**Conclusion:**

The presented benchmark provides a new tool for a statistically unbiased assessment of methods for remote sequence similarity detection, from various complementary perspectives. This tool should be useful both for users choosing the best method for a given purpose, and for developers designing new, more powerful methods. The benchmark set, reference alignments, and evaluation codes can be downloaded from .

## Background

Despite current comprehensive efforts [1,2], three-dimensional structure has been determined for only a fraction of sequence-based protein families [3,4]. Thus there is a need for powerful methods of structure prediction from protein sequence. The most reliable and widely used approaches are based on the detection of statistically supported sequence similarity to proteins of known structure. For both effective development of better structure prediction methods based on sequence similarity, and comparative performance analysis of existing methods, it is crucial to have a good benchmark and a comprehensive methodology for accurate and sensitive evaluation.

The quality of a sequence comparison method can be judged by the quality of 1) similarity detection in a database of sequences or alignments, and 2) sequence alignment for a pair of structurally similar protein domains. These general criteria lead to a variety of possible settings for a method's evaluation. Multiple surveys have been published that compare the performance of methods for remote homology detection and structure prediction (see [5-11] for the recent ones). To assess the quality of similarity detection from sequence, workers generally use a set of protein domains from an established structure classification, such as SCOP [12] or CATH [13], and compare similarity predictions to classification groupings at various hierarchical levels, e.g. family, superfamily, fold, etc. When used as benchmarks, these classifications, while being excellent sources of information, present multiple problems. *First*, although SCOP folds group proteins with general structural similarity and SCOP superfamilies correspond to evolutionary groups, the relationships between superfamilies within the same fold remain undefined: some superfamilies within a fold are very similar and can serve as good templates for each other, others are quite different, and fold similarity between them may not be useful for modeling. *Second*, although many SCOP folds are structurally distinct, there are multiple examples of folds with pronounced structural similarity between them, such as Rossmann-like doubly-wound sandwich folds of α/β SCOP class, Immunoglobulin-like β-sandwiches, and β-propeller folds. *Third*, existing manually curated classifications do not provide reference alignments for the evaluation of sequence-based alignment accuracy. In the published surveys, the accuracy of sequence alignments is usually assessed on a different protein set, by comparing sequence alignments with the 'gold standard' pairwise structure-based alignments produced by some software, for instance DALI [14,15].

In the bi-annual community-wide assessments of protein structure prediction, CASP (Critical Assessment of Techniques for Protein Structure Prediction) [16] and CAFASP (Critical Assessment of Fully Automated Structure Prediction) [17], the testing set is limited to a small number of newly solved protein structures (e.g., 63 proteins in CASP6) [18]. However, along with being a strong catalyst in development of structure prediction methods, CASP pioneered efforts in development of approaches to evaluation of models. Among various parameters used by CASP and related benchmarks, reference-independent global distance test score (GDT_TS) [19,20] has proven to be one of the most informative and robust measures. This score assesses the quality of structure superposition. Large-scale evaluation projects LiveBench [21] and EVA [22] provide quite a few methods to score protein models. However, CASP and LiveBench efforts are largely aimed at structure modeling techniques and thus compare model to structure. Our goal is to validate sequence similarity and homology inference tools, and thus to compare a protein to its homolog or structural analog.

**Figure 1:**
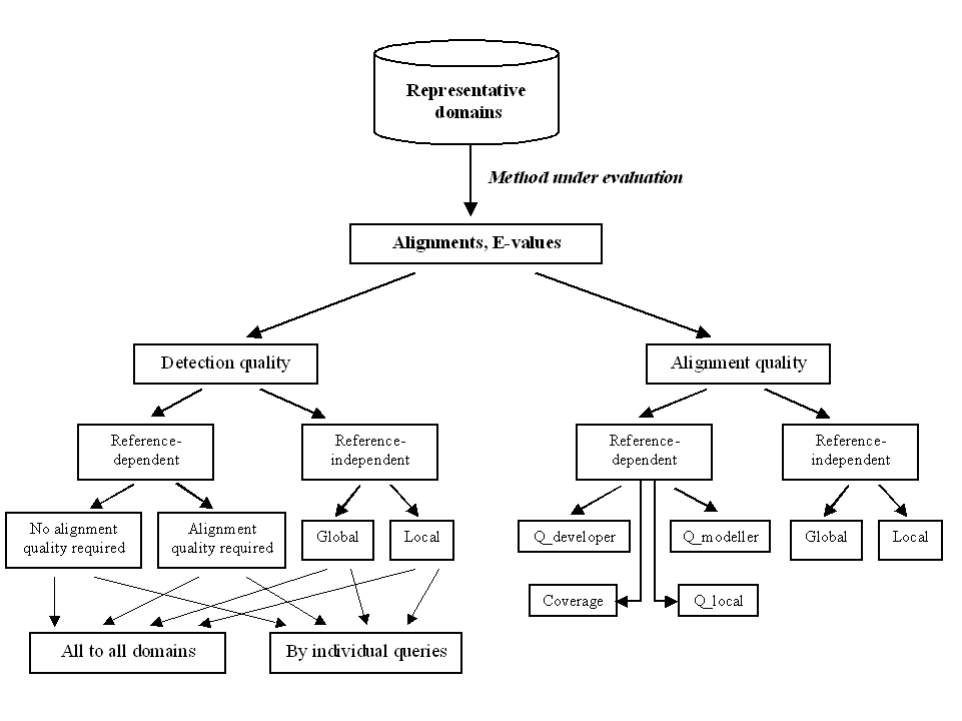
**Workflow of evaluation protocols**. The method under evaluation is run on the diverse set of representative SCOP domains, using each representative as a query for the comparison with the rest domains. The search results are then submitted to the evaluation of the quality of similarity detection ("Detection quality"). This evaluation, based on ROC-like sensitivity curves, is done in both reference-dependent and reference-independent fashion. Reference-dependent evaluation is based on a pre-computed gold standard of similarity relationships between the domains, and on reference (structure-based) alignments. This type of evaluation is performed in two modes that differ in the definition of a true positive hit. The first, more traditional mode ("No alignment quality required"), considers the prediction of similarity regardless of the quality of produced alignment, i.e. a wrong alignment of two similar domains has the same merit as the correct one. The second mode ("Alignment quality required") demands a certain level of alignment accuracy for the hit to be considered a true positive. Reference-independent evaluation does not use any gold standards but is based on the quality of structural superposition suggested by the hit. The quality measures are based on the GDT_TS score and are applied in two modes: global (assessing the superposition over the whole query length) and local (assessing the superposition over the alignment length, however short it is). Both reference-dependent and -independent evaluations are performed on the whole pool of search results ("All-to-all domains"), as well as on the separated results for individual queries ("By individual queries"). The evaluation of alignment quality regardless of assigned statistical significance is performed on the set of structurally similar domains, in both reference-dependent fashion (based on gold-standard structure-based alignments) and reference-independent fashion (based on GDT_TS-like measures for the structure superposition suggested by the evaluated alignment).

To our knowledge, in spite of the variety of existing evaluation protocols [5-11,16,20-22], there is no satisfactory approach that (i) is based on a large and statistically unbiased set of proteins with clearly defined relationships; and (ii) covers all performance aspects of sequence-based structure predictors, such as sensitivity and specificity, alignment accuracy and coverage, and structure template quality. With the aim of designing a comprehensive evaluation method, we (i) select a statistically balanced set of divergent protein domains from SCOP, and define similarity relationships for the majority of these domains with a rigorous algorithm that uses the best of information available in SCOP, while correcting inconsistencies and problems mentioned above; and (ii) develop protocols for the assessment of similarity detection and alignment quality from several complementary perspectives (Fig. 1).

Our protocol is based on three principles. *First*, for a sequence similarity search method to be successful, it needs to (1) rank protein domains from a database in a meaningful way, so that high-scoring domains correspond to good 3D structure matches: i.e. true positives should score high, (2) align those true positives in a way consistent with 3D structure. We refer to (1) and (2) as 'similarity detection' and 'alignment quality' evaluation. *Second*, traditional way of reference-dependent evaluation, in which a ranked hit list or evaluated alignment are compared to a 'gold standard' reference, e.g. to SCOP hierarchy or to DALI alignment, should be complemented with reference-independent evaluation, in which structure comparison characteristics of sequence-based alignment are computed and analyzed. This CASP-style reference-independent evaluation has been successfully applied by us to alignment programs [23,24] and, while showing agreement with reference-dependent evaluation, does not need reference databases, bypassing additional source of problems with reference. *Third*, while there is a need for methods to detect global, domain-level similarity, in particular for finding good templates for structure modeling and evolutionary links between domains, it is of an interest to find short, local fragments that are conformationally similar to fragments of the query domain. These fragments can be used by structure prediction methods to assemble models from. We evaluate both 'global' and 'local' properties of a search algorithm.

General workflow of the evaluation protocol that we have developed is shown on Fig. 1. *First*, we assess the quality of similarity detection, i.e. method's ability to discriminate between correct and incorrect similarity predictions by estimating their statistical significance. ROC curves [25] and sensitivity curves similar to ROC curves are constructed, based on several criteria for a match to be true positive. Reference-dependent evaluations use a 'gold-standard' set of pre-defined relationships between domain pairs and a set of structural alignments. The more traditional of these protocols regards as correct any prediction of similarity for domains that are known to be similar; the other, more stringent protocol additionally requires a certain degree of alignment accuracy (Fig. 1). Reference-independent evaluations are based on GDT_TS scores and thus inherently take into consideration the quality of predicted alignments. In these protocols, GDT_TS scores are adjusted for the global and local modes of evaluation (Fig. 1). The global mode favors fold predictions for the whole query domain and tolerates potential inaccuracy of detail. The local mode rewards precision of the predicted alignment, however short the predicted fragment is, while correcting for higher probability of a good quality random match between shorter fragments. There might be a situation when the estimates of statistical significance produced by a method from a raw alignment score are biased by the query's length, amino acid composition, number of sequences in an alignment and other factors. This bias would deteriorate the performance on the set of all-to-all domain comparisons, in spite of a good ranking of hits by the raw score for each individual query. In order to control for such situation, each protocol includes both assessment of all-to-all comparisons combined within the dataset, and the evaluation of detection quality for individual query domains (Fig. 1). *Second*, we assess the produced alignments of true positives regardless of their assigned statistical significance. For pairs of similar domains binned by sequence identity, we calculate several reference-dependent and reference-independent measures of alignment quality (Fig. 1).

We apply this evaluation pipeline to several well-known sequence and profile methods and show that different aspects of evaluation (e.g. global vs. local, or combined all-to-all vs. individual query) reveal different properties in the evaluated methods, highlighting advantages and weaknesses of each method.

## Results and discussion

To assess a method for remote sequence similarity detection (Fig. 1), we evaluate the method as 'a searcher' (by the quality of ranking the produced predictions) and as 'an aligner' (by the quality of constructed alignments). First, we carefully select a dataset of 4000 domain representatives from SCOP, and determine their similarity relations by combining the expert SCOP assignments of superfamilies with multiple automated methods for structure and sequence comparison. We are able to reduce the number of 'undefined' domain relationships to 10% of the whole set, and to re-define similarity relations between some of the SCOP folds. Second, we use the pairs of similar domains as a benchmark for the assessment of alignment quality, from both global (whole domain length) and local (aligned regions) perspectives. Third, we use all-to-all comparisons in the whole set of domains to assess the quality of similarity detection. We develop four protocols with different definitions of true and false positive predictions (Fig. 1). As an illustration, we benchmark several profile-based methods for sequence comparison and compare their performance from several different standpoints.

### Benchmark dataset

**Figure 2:**
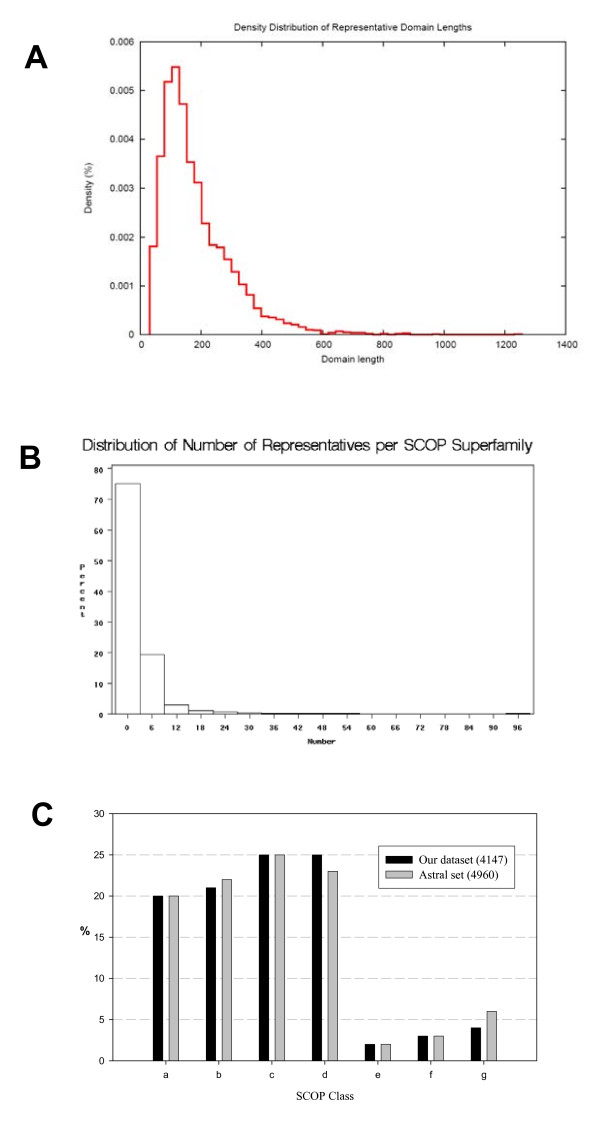
**Distributions of properties of representative domains**. A. Distribution of domain lengths. B. Distribution of number of representatives per SCOP superfamily. C. Distribution of number of representatives per SCOP class. Black bar shows the distribution of our representative dataset (4147 domains). Gray bar shows the distribution of Astral SCOP20 set (4960 domains). The x-axis shows the abbreviate names of the SCOP classes. a: all alpha proteins; b: all beta proteins; c: alpha/beta proteins; d: alpha + beta proteins; e: multi-domain proteins; f: membrane and cell surface proteins and peptides; g: small proteins.

Our benchmark set of protein domains (see Additional File 1) (i) comprises a large statistical sample; (ii) provides unbiased representation of existing protein structures; and (iii) contains a considerable portion of domains pairs whose similarity is challenging to detect. The dataset contains 4147 SCOP domains from 1516 superfamilies of seven classes. Fig. 2A shows the distribution of domain lengths, between 31 and 1256 residues with a median of 151 residues. Fig. 2B shows the distribution of number of representatives per SCOP superfamily. Representation of different SCOP classes in our dataset is similar to the Astral SCOP20 set (Fig. 2C).

SCOP is a manually curated structure classification based on both structural and evolutionary considerations. Well known for a high quality of domain delineation and diligent analysis of domain similarities, SCOP is often used as a 'gold standard' for homology detection and fold recognition [26-28]. However, there are at least two factors that limit the quality of this classification as a standard: a large 'gray area' of unknown relationships between different superfamilies of the same SCOP fold, and many examples of different folds that are structurally similar to the extent that one would serve as a good template for another. The simplest solution is to regard only well-established categories, considering domains from the same superfamily as similar, domains from different classes as dissimilar, and disregarding all intermediate cases (same fold or same class) as 'unknowns' [29]. However, in a set of SCOP domain representatives, the 'unknown' category may include a considerable fraction of pairs. To reduce this category, Soding [29] applied MaxSub score [30] as an additional criterion of domain similarity.

Our goal was to use the well-established (but often intricate) cases of similarity represented by SCOP superfamilies, on one hand, and the clear cases of dissimilarity represented by different SCOP classes, on the other hand, and to train SVM on these cases. The resulting SVM does not reproduce criteria of SCOP classification, but is directed only at the structural similarity between domains. As detailed below, application of this SVM adds a significant number of structural similarities that are generally consistent with multiple observations of different research groups. Similar SVM-based approaches (train on a clear-cut SCOP subset and apply to all SCOP domains) have been successfully used by others (e.g. [31]).

In order to produce statistically sound benchmark dataset, we set the goal of reducing the fraction of 'unknown' relationships to as low as 10%. To achieve this goal, we use an SVM-based consensus of a number of structure- and sequence-based methods, which allows us to classify the majority of 'unknown' SCOP category. Out of total 17,197,609 domain pairs, only 123,185 share a SCOP fold, whereas 483,474 are classified as similar by SVM. SVM more than triples the number of similar domains, adding 377,374 similar pairs from different folds. About two thirds of these (225,167 pairs) have DALI Z-score > 3.0; the inclusion of the remaining pairs was guided by other SVM features, mainly FAST score, the second largest SVM component. Distribution of the added pairs among SCOP classes reflects the structural diversity within each class and their representation in the whole set. About a half (196,596 pairs) belongs to the large α/β class, which contains many homologous Rossmann-type folds that are separated in SCOP by features other than overall structural similarity [32]. Out of 136 structural folds in the α/β class of SCOP 1.69, at least 77 folds are Rossmann-type, with a structure pattern of the doubly-wound α/β sandwich. Next large group (60688 pairs) belongs to all-alpha class containing a number of structurally similar folds. Many domains of all-beta class (32269 pairs) belong to multiple immunoglobulin sandwich folds and beta-propeller folds. α + β class is the most diverse and accordingly the least represented of the major SCOP classes: it contributes 25628, or ~ 7% of all similar domain pairs added by SVM. At the same time, only 17,085 pairs that share a fold are not classified as similar by SVM. On the SCOP superfamily level, only 49,077 pairs should be classified as similar, almost 10 times fewer than classified by SVM. Out of total 17,197,609 domain pairs, our method classifies 483,474 pairs (3%) as similar ('true'), 14,961,720 pairs (87%) as dissimilar ('false'), and 1,752,415 pairs (10%) as 'unknown'.

Creating a reliable benchmark set of pairwise similarities between domains is different from the task of domain classification. The problem of automated classification of SCOP domains has been addressed elsewhere (e.g. [31,33]). Our goal was not to construct a new classification, but to assign more consistent similarity relationships to domain pairs for benchmarking purposes. Classification would further require establishing transitive connections between domains and additional optimization of the methods for hierarchical grouping of domains, which is out of scope of this work.

**Figure 3:**
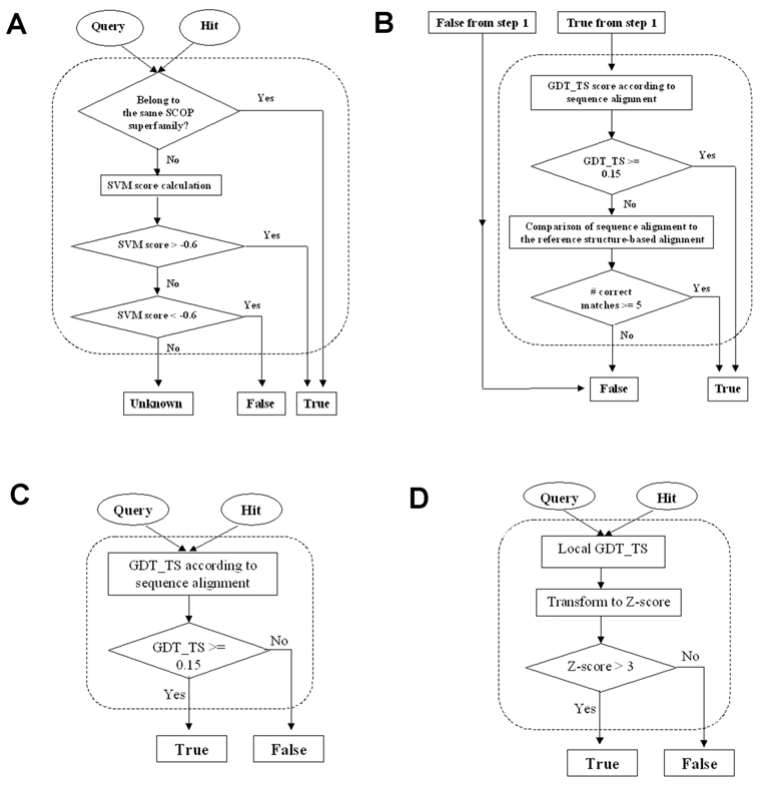
**Flowcharts of evaluation of similarity detection**. A. Reference-dependent, with no consideration of alignment quality. B. Reference-dependent, with the requirement of alignment quality for true positive hits. C. Reference-independent, global mode. D. Reference-independent, local mode.

Reference-dependent evaluation of method's performance can be performed with two types of reference: (i) known relationships between domains of the testing set; and (ii) known structure-based alignments of domain sequences. In fold recognition assessment, evaluation (i) has been traditionally performed using a large-scale structure classification (SCOP or CATH), whereas evaluation (ii) has been ignored. In other words, a true similarity prediction providing incorrect alignment has been treated the same as a prediction providing correct alignment. In our benchmark, this traditional approach (Fig. 3A) is complemented by the alignment-based approach, where the true predictions of domain similarity are additionally assessed by the criteria of alignment quality (Fig. 3B, see Methods). There are two main motivations behind this approach. First, better alignment quality often results in better recognition of sequence similarity. For example, results of recent CASPs (e.g. [16]) show a need for such a combined evaluation of structure modeling methods. Second, a better method may consist of two separate steps: initial detection of a similarity followed by the construction of a high-quality alignment for the detected pair. Put in a pipeline, these steps may produce a better structural model. Our evaluation criteria would cover this case as well.

Both types of reference-dependent criteria would penalize short alignments that are structurally reasonable but differ from the 'gold standard', for example, alignments of similar structural fragments (beta-hairpins, etc.) Therefore reference-dependent evaluation may be considered a global rather than local assessment of structure prediction.

In addition, we perform evaluations that do not require any prior knowledge about protein relationships or alignments. These evaluations are based on the quality of structure superposition suggested by a sequence alignment of interest, reflected in the GDT_TS score [19,20]. The global mode (Fig. 3C) rewards detecting good overall structural templates for the query, i.e. global fold recognition. The local mode (Fig. 3D) rewards finding local but precise alignments to segments of the query, i.e. fragment modeling. Different sequence-based alignment methods may be optimal for different modes of structure prediction. Thus using both modes is necessary for a balanced and informative evaluation.

### Evaluation examples

As an illustration, we use our benchmark to compare the performance of several methods for the detection of remote sequence similarities: PSI-BLAST version 2.2.1 [34], methods for profile-profile comparison (COMPASS version 1.24 [35], version of HHsearch [29] with no secondary structure (SS) information used), and for the comparison of profiles combined with predicted SS (HHsearch [29], prof_ss [26]). These programs are used for all-against-all comparison of domains in the benchmark set. The resulting sequence alignments, with statistical significance assigned, are processed using the evaluation methods described above.

**Figure 4:**
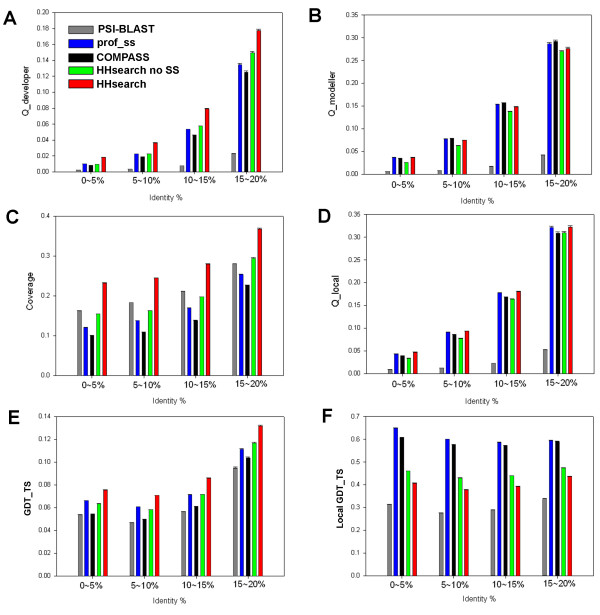
**Alignment quality for profile-based methods of sequence comparison**. Reference-dependent parameters (Q_developer _(A), Q_modeller _(B), coverage (C), Q_local _(D)) and reference-independent parameters (original GDT_TS (E) and local GDT_TS version (F)) are compared for PSI-BLAST, COMPASS, prof_ss, and HHsearch versions with and without consideration of predicted secondary structure, for several bins of sequence identity.

For the assessment of alignment quality, all pairs of domains defined as similar are used, and various parameters of the produced alignments are calculated for several ranges of sequence identity (Fig. 4). For each parameter, the rankings of methods are consistent in all identity bins. Despite similar alignment coverage (Fig. 4C), profile-profile methods in general achieve alignment accuracy higher than PSI-BLAST, which involves profile-sequence comparison (Fig. 4A,B, D–F).

The results of reference-dependent assessment are shown in Fig. 4A–D. HHsearch, a method utilizing SS predictions for the compared profiles [29], produces the largest number of accurate residue matches reflected in Q_developer _(Fig. 4A), whereas COMPASS and prof_ss provide a larger fraction of accurate matches within the produced alignment, as reflected in Q_modeller _(Fig. 4B). These differences seem to be caused mainly by the higher coverage achieved by longer HHsearch alignments (Fig. 4C). The local accuracy over the aligned structural regions is much more similar for all the methods (Fig. 4D), although Q_local _is slightly higher for the methods that consider SS predictions, HHsearch and prof_ss.

The results of reference-independent assessment of alignment quality are shown in Fig. 4E,F. The original GDT_TS scores are shown in Fig. 4E. HHsearch performs best, followed by prof_ss and profile-profile methods with no SS considered. This result is similar to the global reference-dependent evaluation by Q_developer _(Fig. 4A). The ranking by local GDT_TS measure (Fig. 4F) is different from the global measure: prof_ss shows the best performance, closely followed by COMPASS, in accord with the ranking by reference-dependent local measure Q_modeller _(Fig. 4B). Notably, HHsearch version with SS considered produces lower local GDT_TS than the version without consideration of SS (Fig. 4F), presumably due to the inclusion of alignment regions with higher structural deviation between domains.

**Figure 5:**
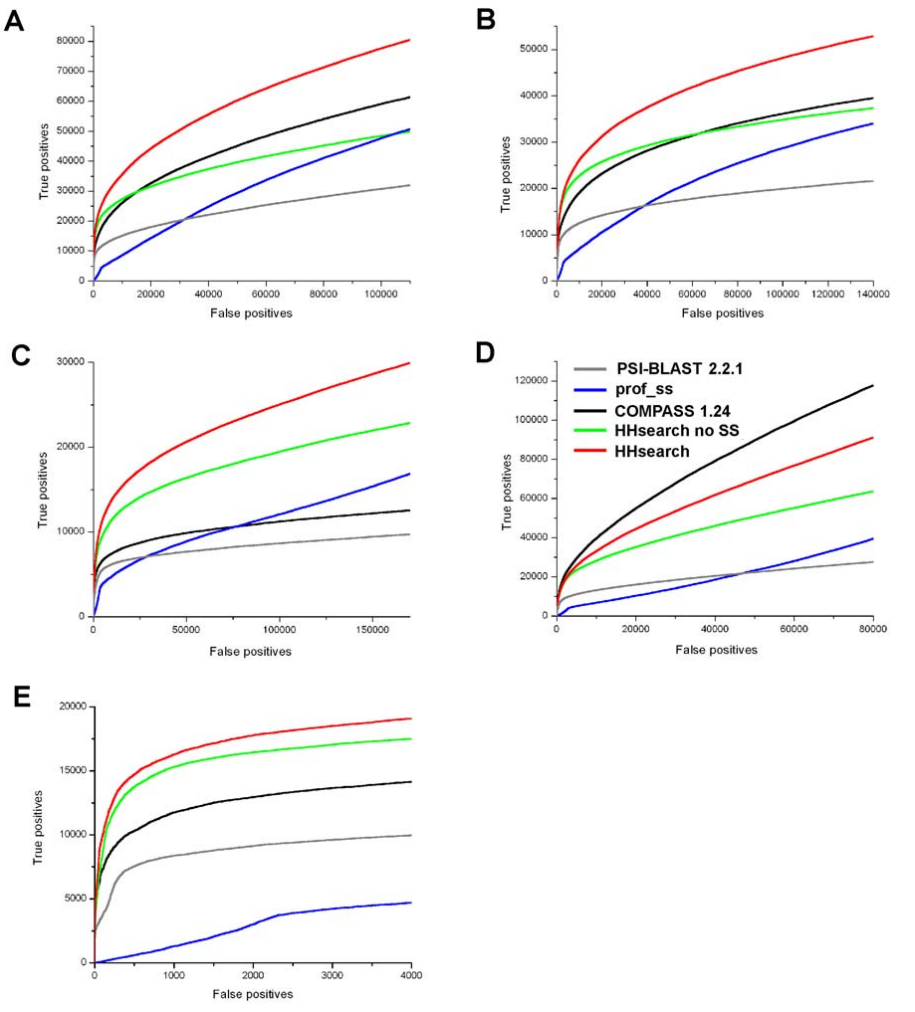
**Quality of similarity detection for profile-based methods of sequence comparison, with different criteria for true positive hits**. A. Reference-dependent without consideration of alignment quality. B. Reference-dependent with additional requirement for a minimal alignment quality. C. Reference-independent, global mode. D. Reference-independent, local mode. E. Criteria based on SCOP only: domains in the same superfamily are considered true, domains in different classes are considered false, domains in the same class but different superfamilies or folds are considered 'unknown' and disregarded.

For the evaluation of sequence similarity detection, we use sensitivity curves similar to ROC curve [25], which are based on the list of predictions (hits) produced by a method of interest and sorted by their statistical significance (e.g. by ascending E-value). We construct several sensitivity curves based on different approaches to the assessment of hits as true or false positives (Fig. 5). In all evaluation settings, methods based on profile-profile comparison show better performance than PSI-BLAST. HHsearch performs best in three of the four settings. Profile-profile methods with no SS consideration are ranked next in most cases.

Fig. 5A and 5B respectively show the results of reference-dependent evaluation with no requirement for alignment quality, and with a certain level of alignment accuracy required for a hit to be considered a true positive. Notably, the two evaluations produce the same ranking of methods' performance. According to reference-dependent assessment, HHsearch provides the best similarity detection, followed by COMPASS and the version of HHsearch with no SS considered, then prof_ss and PSI-BLAST. Thus additional analysis of accuracy of predicted alignments does not make significant changes in the ranking of evaluated methods. This result suggests that the quality of predicted alignment generally correlates with statistical significance of a hit, and that all methods produce similar fractions of the top true positive hits with accurate alignments.

Fig. 5C and 5D show the results of reference-independent evaluation in local (Fig. 5C) and global (Fig. 5D) modes. Interestingly, the ranks of methods in the global mode are similar to the reference-dependent setting, except for a clearly lower performance of COMPASS compared to the HHsearch version with no SS considered. This difference may be attributed to the length of the alignments produced by the two methods. According to our observations, COMPASS tends to construct shorter alignments including regions of highest similarity between two profiles, whereas HHsearch generates alignments covering longer segments of the query. Despite the latter alignments may include regions of lower accuracy, they are more valuable for the prediction of whole domain structure and therefore favored by the global evaluation mode (Fig. 5C). The difference in the alignment coverage of the two methods may be due to the different methodological frameworks. COMPASS employs the 'traditional' profile comparison and uses Smith-Waterman algorithm for the alignment construction with fixed gap penalties, whereas HHsearch is based on HHM-HHM comparison and features adjustable gap penalties, which might produce more extended alignments.

In the local mode of reference-independent evaluation (Fig. 5D), the methods are ranked differently: the performance of COMPASS is the best, followed by the performance of HHsearch versions with and without consideration of SS. This ranking suggests that shorter alignments produced by COMPASS have higher prediction accuracy 'per residue' and may provide a better detection of local structural similarities. Comparison of Fig. 5C and 5D illustrates a tradeoff between the quality of global and local predictions: more extended alignments tend to include a larger portion of less accurate regions but better predict overall structural fold, whereas shorter alignments limit the ability for global fold recognition but are better in precise modeling of structural fragments.

Fig. 5E shows the sensitivity curves produced in the same setting as in Fig. 5A–D, but with true and false positives determined by SCOP relations only (true, same superfamily; false, different classes). The relative difference between the two HHsearch versions is much less pronounced than on our benchmark, since the addition of secondary structure gains the main improvement in the area of extremely remote sequence similarity that corresponds to SCOP levels higher than superfamily (i.e. domains from different SCOP superfamilies and folds).

The results of paired tests for detection quality on individual queries (Table 1) are different from the results on all-to-all domain comparisons. For reference-dependent evaluation without consideration of alignment quality of the produced hits, the ranking of performance is HHsearch > HHsearch (no SS) > prof_ss > COMPASS > PSI-BLAST, where '>' sign denotes statistically significant difference (Table 1). Consideration of alignment quality results in the same ranking. The ranking produced by the global mode of reference-independent evaluation is HHsearch > COMPASS > HHsearch (no SS) > prof_ss > PSI-BLAST, whereas the ranking for the local mode is prof_ss > HHsearch > COMPASS > HHsearch (no SS) > PSI-BLAST (Table 1).

**Table 1:** Paired tests for detection quality on individual queries

	**PSI-BLAST**	**COMPASS**	**HHsearch no SS**	**prof_ss**	**HHsearch**
**PSI-BLAST**					
0.09		-0.00	-6.39e-317	-0.00	-0.00
0.18		-0.00	-0.00	-1.03e-317	-0.00
0.31		-2.85e-179	-2.71e-248	-2.19e-180	-1.34e-276
0.12		-0.00	-0.00	-0.00	-0.00
**COMPASS**					
0.13	+0.00		-2.96e-41	-8.63e-200	-0.00
0.22	+0.00		-3.48e-151	-8.48e-48	-0.00
0.38	+2.85e-179		+4.10e-07	+3.89e-22	-1.18e-52
0.24	+0.00		+4.75e-141	-0.00	-0.00
**HHsearch no SS**					
0.14	+6.39e-317	+2.96e-41		+0.00	-0.00
0.27	+0.00	+3.48e-151		+6.92e-10	-2.60e-274
0.38	+2.71e-248	-4.10e-07		+4.44e-22	-5.06e-139
0.22	+0.00	-4.75e-141		-0.00	-0.00
**prof_ss**					
0.14	+0.00	+8.63e-200	-0.00		-0.00
0.21	+1.03e-317	+8.48e-48	-6.92e-10		-2.77e-258
0.37	+2.19e-180	-3.89e-22	-4.44e-22		-1.76e-15
0.45	+0.00	+0.00	+0.00		+0.00
**HHsearch**					
0.19	+0.00	+0.00	+0.00	+0.00	
0.28	+0.00	+0.00	+2.60e-274	+2.77e-258	
0.38	+1.34e-276	+1.18e-52	+5.06e-139	+1.76e-15	
0.34	+0.00	+0.00	+0.00	-0.00	

Methods are compared by sensitivity values at 50% selectivity, calculated separately for each query. For each pair of methods, P-values of paired Wilcoxon rank test are shown for four types of criteria for true/false positive distinction (from top to bottom): reference-dependent without alignment quality and with alignment quality, and reference-independent global and local. Plus and minus signs by the P-values denote respectively positive and negative difference for the method indicated on the left Vs the method indicated on the top. The left column includes mean values over all queries for each method.

The assessment of detection quality split into individual queries disregards the ability of a method to make the estimates of statistical significance that would be transferable between queries. In this assessment, only ranks of hits produced by individual query are considered. Typically, for a given query the estimated statistical significance of a hit (e.g. E-value) monotonically depends on the 'raw' score of the produced alignment. Therefore, for the hits produced by a single query the ranks of E-values are the same as the ranks of raw scores. Thus splitting the assessment into individual queries in effect bypasses the procedure of estimating statistical significance altogether. Comparing the results of this assessment (Table 1) to the results for all queries combined (Fig. 5) highlights the quality of the E-value estimation implemented by different methods. In particular, the evaluation on individual queries (Table 1) shows higher performance for the methods using SS predictions (prof_ss in local reference-independent mode and HHsearch in all other modes), which suggests better ranking by the raw alignment scores when a single query is considered. In contrast, the performance of prof_ss is lower according to 'all-to-all' evaluations (Fig. 5). These results suggest that prof_ss is more applicable to the search for locally similar structural fragments, and that this method may have a potential to improve the estimates of statistical significance, so that these estimates are more compatible for different queries, regardless of query's length, residue composition, and other properties.

The results produced by the presented benchmark are in general accord with previously reported evaluations [7,8,29] in the parts where similar approaches are used. Our results confirm the superiority of profile-profile comparison over profile-sequence comparison. In addition, both our reference-dependent evaluation (Fig. 5A,B) and reference-independent evaluation (Fig. 5C,D) support the conclusion [29] that the use of predicted secondary structure in the comparison of MSAs can improve the quality of similarity detection. In particular, HHsearch version that uses secondary structure performs better than HHsearch without the use of secondary structure (Fig. 5A–D). Compared with benchmarks that are based entirely on SCOP (see Fig. 5E as an example), our testing set provides much higher sensitivity in the distinction between different methods, mainly due to a careful selection of divergent domains and a small fraction of 'unknown' relationships.

We also introduce evaluation approaches that have not been used in similar systems before. In some cases, the results produced by these approaches differ from the results of more traditional evaluations. First, we provide GDT_TS-based reference-independent assessments of similarity detection (Fig. 5C,D). The two modes of this evaluation, global and local, provide additional information about the quality of the searches for the overall structure similarity between domains and for similar structural fragments, respectively. As in the case of COMPASS 1.24 (Fig. 5C,D), the same method can perform quite differently according to these two types of detection quality. Such evaluations allow the user to determine which methods are more appropriate for a particular task. Second, in addition to the evaluations of similarity detection on the whole set of all-to-all domain comparisons, we implement the separate evaluation for different queries. A similar approach was proposed by Soding [29] who constructed the distributions of MaxSub scores of the top hit for each query. We use a more direct approach that is based on individual sensitivity/selectivity curves and provides a single number (P-value) characterizing the comparison between two detection methods. Such evaluation should be especially valuable for developers of new methods, allowing them to evaluate other parts of the methods separately from the tedious and often problematic procedure of E-value estimation. As an example, this evaluation (Table 1) shows a potential for further development of prof_ss and pinpoints the procedural part that may cause a lower performance of this method in the all-to-all setting (Fig. 5).

## Conclusion

We have developed a comprehensive benchmark system that serves as a tool for a statistically unbiased assessment of methods for remote sequence similarity detection, from various complementary perspectives. This tool should be useful both for users choosing the best method for a given purpose, and for developers designing new, more powerful methods. The benchmark set, reference alignments, and evaluation codes are available to download from [36].

## Methods

The work includes three parts: (i) delineation of a large, statistically balanced testing set of protein domains; (ii) protocols for the evaluation of similarity detection; and (iii) protocols for the evaluation of alignment quality. Accordingly, we *first *construct a dataset of diverse representative SCOP domains and define the similarity relationships between them. Our aim is to overcome drawbacks of SCOP as a reference for such relationships, namely to reduce the number of undefined relationships between domains that belong to different superfamilies of the same fold, and to establish similarities between related folds. To define the domain relationships, we complement the SCOP superfamily classification with SVM-optimized linear combination of several structure- and sequence-based similarity scores. *Second*, we develop the protocols for the evaluation of similarity detection. Each protocol processes the list of alignments sorted by their statistical significance and produces a sensitivity, ROC-like curve based on a corresponding definition of true/false positives. To define true and false positives, we implement both reference-dependent and reference-independent approaches (Fig. 1). Similarity detection evaluations are carried out both on the combined list of all-to-all domain comparisons, in order to assess the overall method performance; and on the separate lists of alignments for each domain, in order to assess the method's performance on individual queries. *Third*, we develop the protocols for the assessment of alignment quality, regardless of estimated statistical significance. Based on the domain pairs defined as similar, we calculate several measures that reflect alignment coverage and accuracy, with and without respect to the reference structure-based alignments (Fig. 1).

### 1. Benchmark protein set

To produce a testing set that would present a considerable challenge for sequence similarity detection, we focused on fairly low cutoffs of sequence identity between individual domains (15–25%). Astral Compendium [37,38] provides representatives of SCOP [12] with high-quality structures for various identity cutoffs. However, the identity in Astral is derived from sequence-based alignments that could be inaccurate at this level of sequence similarity. Therefore, we started from ASTRAL representatives at 40% cutoff, and used their structure-based alignments to select the set with lower maximal identity. To ensure the accuracy of the results, we combined three different structural alignment methods, DALI [14], TM [39] and FAST [27].

#### Methods for pairwise identity calculation

To probe the influence of gaps and unaligned regions in the structure-based alignments, we tested three methods of calculating sequence identity: (i) percentage identity within aligned blocks only, pid(1)=NidLali
 MathType@MTEF@5@5@+=feaafiart1ev1aaatCvAUfKttLearuWrP9MDH5MBPbIqV92AaeXatLxBI9gBaebbnrfifHhDYfgasaacH8akY=wiFfYdH8Gipec8Eeeu0xXdbba9frFj0=OqFfea0dXdd9vqai=hGuQ8kuc9pgc9s8qqaq=dirpe0xb9q8qiLsFr0=vr0=vr0dc8meaabaqaciaacaGaaeqabaqabeGadaaakeaacqWGWbaCcqWGPbqAcqWGKbazcqGGOaakcqaIXaqmcqGGPaqkcqGH9aqpdaWcaaqaaiabd6eaonaaBaaaleaacqWGPbqAcqWGKbazaeqaaaGcbaGaemitaW0aaSbaaSqaaiabdggaHjabdYgaSjabdMgaPbqabaaaaaaa@3DD4@, where *N*_*id *_is the number of aligned identical residues, *L*_*ali *_is the length of the aligned regions; (ii) percentage identity over the shorter sequence, pid(2)=NidLshorter
 MathType@MTEF@5@5@+=feaafiart1ev1aaatCvAUfKttLearuWrP9MDH5MBPbIqV92AaeXatLxBI9gBaebbnrfifHhDYfgasaacH8akY=wiFfYdH8Gipec8Eeeu0xXdbba9frFj0=OqFfea0dXdd9vqai=hGuQ8kuc9pgc9s8qqaq=dirpe0xb9q8qiLsFr0=vr0=vr0dc8meaabaqaciaacaGaaeqabaqabeGadaaakeaacqWGWbaCcqWGPbqAcqWGKbazcqGGOaakcqaIYaGmcqGGPaqkcqGH9aqpdaWcaaqaaiabd6eaonaaBaaaleaacqWGPbqAcqWGKbazaeqaaaGcbaGaemitaW0aaSbaaSqaaiabdohaZjabdIgaOjabd+gaVjabdkhaYjabdsha0jabdwgaLjabdkhaYbqabaaaaaaa@439C@, where *L*_*shorter *_is the length of the shorter of the two sequences; (iii) identity within the aligned blocks combined with a background identity for the unaligned regions, pid(3)=Nid+Lunali∗pidrandomLshorter
 MathType@MTEF@5@5@+=feaafiart1ev1aaatCvAUfKttLearuWrP9MDH5MBPbIqV92AaeXatLxBI9gBaebbnrfifHhDYfgasaacH8akY=wiFfYdH8Gipec8Eeeu0xXdbba9frFj0=OqFfea0dXdd9vqai=hGuQ8kuc9pgc9s8qqaq=dirpe0xb9q8qiLsFr0=vr0=vr0dc8meaabaqaciaacaGaaeqabaqabeGadaaakeaacqWGWbaCcqWGPbqAcqWGKbazcqGGOaakcqaIZaWmcqGGPaqkcqGH9aqpdaWcaaqaaiabd6eaonaaBaaaleaacqWGPbqAcqWGKbazaeqaaOGaey4kaSIaemitaW0aaSbaaSqaaiabdwha1jabd6gaUjabdggaHjabdYgaSjabdMgaPbqabaGccqGHxiIkcqWGWbaCcqWGPbqAcqWGKbazdaWgaaWcbaGaemOCaiNaemyyaeMaemOBa4MaemizaqMaem4Ba8MaemyBa0gabeaaaOqaaiabdYeamnaaBaaaleaacqWGZbWCcqWGObaAcqWGVbWBcqWGYbGCcqWG0baDcqWGLbqzcqWGYbGCaeqaaaaaaaa@5A28@, where *L*_*unali *_is the length of the unaligned regions in the shorter sequence, and *pid*_*random *_is the background identity of random alignments. We estimated the background identity as pidrandom=∑i=120fi2
 MathType@MTEF@5@5@+=feaafiart1ev1aaatCvAUfKttLearuWrP9MDH5MBPbIqV92AaeXatLxBI9gBaebbnrfifHhDYfgasaacH8akY=wiFfYdH8Gipec8Eeeu0xXdbba9frFj0=OqFfea0dXdd9vqai=hGuQ8kuc9pgc9s8qqaq=dirpe0xb9q8qiLsFr0=vr0=vr0dc8meaabaqaciaacaGaaeqabaqabeGadaaakeaacqWGWbaCcqWGPbqAcqWGKbazdaWgaaWcbaGaemOCaiNaemyyaeMaemOBa4MaemizaqMaem4Ba8MaemyBa0gabeaakiabg2da9maaqahabaGaemOzay2aa0baaSqaaiabdMgaPbqaaiabikdaYaaaaeaacqWGPbqAcqGH9aqpcqaIXaqmaeaacqaIYaGmcqaIWaama0GaeyyeIuoaaaa@456C@, where *f*_*i *_is the background frequency of amino acid type *i*. Background residue frequencies either estimated by Dayhoff [40] or derived from the SCOP 40% representatives produce the estimate of *pid*_*random *_= 6%, consistent with our observations of unaligned regions in DALI alignments.

#### Selecting domain representatives

To ensure that the dataset (i) includes representatives from each SCOP superfamily; and (ii) contains the largest possible number of domains, we use a novel method of domain selection. In each SCOP superfamily, we use a dynamic programming algorithm to select the largest number of domain representatives with sequence identity below a given cutoff.

**Figure 6:**
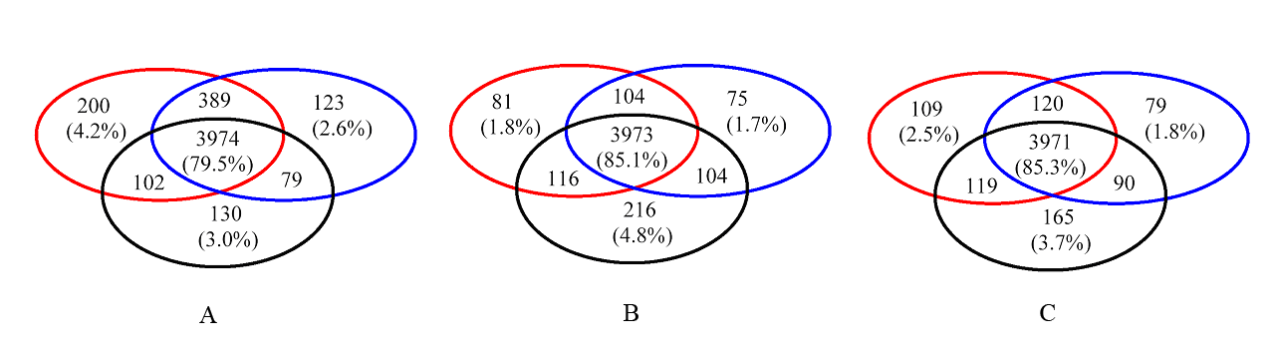
**Domain representatives selected using different structure alignment programs and different identity measures**. Red circle: TM alignment-based representatives. Blue circle: DALI alignment-based representatives. Black circle: FAST alignment-based representatives. A. Representatives selected using *pid*(1). B. Representatives selected using *pid*(2). C: Representatives selected using *pid*(3). See text for details.

Such representative sets are selected at various identity cutoffs, for each of the alignment methods (DALI, TM, and FAST) and each of the identity measures used (*pid*(1), *pid*(2), and *pid*(3)). Our assumption is that the best measure of sequence identity should most closely reflect the actual evolutionary distance between the domains, regardless of the specific differences in the alignment methods. Thus we look for the measure that provides the largest overlap between representatives produced by all three aligners, and the smallest number of unique representatives produced by only one aligner and not by others. Screening the identity cutoffs in the range of 15–25%, we find that the size of the overlapping representative set is about 3500–4500 representatives for all *pid *measures. Fixing this size to approximately 4000, which corresponds to the sequence identity of ~ 20%, we compared the identity measures by the number of generated unique representatives. Fig. 6 shows Venn diagrams of representatives produced by the three aligners, using measures *pid*(1) (A), *pid*(1) (B), and *pid*(1) (C). The simplest identity measure *pid*(1) is clearly the worst, producing the largest number of unique representatives. The total numbers of unique representatives for *pid*(2) and *pid*(3) are lower. As the best measure, we choose *pid*(3), because it (i) produces the lowest average number unique representatives (2.7%); and (ii) unlike *pid*(2), generates a similar number of representatives for each aligner. Thus, for the final dataset, we select the overlap between the representatives produced by all three aligners using identity measure *pid*(3) (Fig. 6C).

Multiple sequence alignments for each sequence in the representative dataset are generated using PSI-BLAST with conservative settings (inclusion E-value cutoff of 10^-4 ^for up to 2 iterations). Secondary structures for each of the representative sequence are predicted using PSIPRED [41]. Compared to real secondary structures generated by DSSP [42] based on the 3-dimensional structures of the domains, the average accuracy of the predicted secondary structure is 80% for Q3 [43] and 78% for SOV [44], which are similar to reported prediction accuracy for PSIPRED [45].

### 2. Evaluation of similarity detection

To evaluate the quality of similarity detection by the method of interest, we use (i) sensitivity curves based on all-to-all comparisons in the benchmark domain set; and (ii) paired comparison between the methods by sensitivity/selectivity measures for individual queries from this set. Sensitivity curves are based on the list of alignments (hits) sorted by their statistical significance, with the most significant hits on top. Each hit is assessed as true or false positive by a corresponding protocol as described below, and the number of true positives is plotted against the number of false positives, starting from the top of the list. Different protocols for the assessment of predictions produce different assignments of true/false positives, resulting in different curves. Such evaluation of all-to-all domain comparisons might be biased if a few query domains produce many highly significant hits that dominate the top of the hit list. Hence, in addition we compare the quality of detection query by query. We construct sorted lists of hits for each query domain, and consider sensitivity (*sensitivity *= TP/(TP+FN), where TP and FN are the numbers of true positives and false negatives, respectively) at a given level of selectivity (*selectivity *= TP/(TP+FP), where FP is the number of false positives). These sensitivity values for the evaluated methods are compared using paired t-test and non-parametric paired Wilcoxon rank test. We find that 50% level of selectivity reveals the most significant differences between the compared methods, and that results are similar for the t-test and Wilcoxon test.

#### 2.1 Reference-dependent evaluation of similarity detection

According to our definition, reference-dependent evaluation (Fig. 3A,B) uses information about structure classification (e.g. SCOP or other sources) and structure-based alignments, which are assumed to be correct.

The first traditional step in the evaluation is to assign each pair of proteins that are being compared to one of the two classes: true of false. We term this 'template quality' evaluation. Pairs from the 'true' class are structurally similar and could serve as templates for each other in homology modeling. This assignment is a very difficult task. Due to imperfection in our knowledge and in automatic methods, it has been suggested that it may be beneficial to avoid making this decision in difficult cases and to set aside the pairs of 'undefined' quality thus not using them in evaluation [7,29]. Such difficult pairs are usually those with marginal structural similarity. Our aim was to reduce the number of 'undefined' relationships among the selected SCOP domains to as low as 10% of the total, so that these domains do not introduce significant bias in the evaluation results. To minimize the number of 'undefined', we designed a sensitive method, which combines both structural and sequence similarity measures. Combined with SCOP classification, this method allows us to (i) clearly define relationships between the majority of domains in the same SCOP fold but in different superfamilies; and (ii) in many cases, establish similarity between domains that belong to different SCOP folds, but could serve as good templates for each other. Using this method, we attribute each pair to one of the three classes: true, false and undefined (Fig. 3A), and evaluate performance of sequence searchers with ROC curves [25]. Pairs from undefined category are set aside and do not participate in the evaluation.

At the second step (Fig. 3B), we additionally impose the requirements on alignments produced by a sequence searcher. For the classic ROC curve, the accuracy of sequence alignments is not checked, so any match of a 'true' pair is considered 'true'. Alignment accuracy criteria reduce the number of 'true' pairs by considering the pairs with incorrect alignments as 'false'. This additional requirement is more meaningful in terms of structure modeling, but since assignment to 'true' or 'false' category depends on the sequence alignment, and thus on the method being tested, a pair can be 'true positive' for one method, and 'false positive' for another method. Therefore the evaluation curves do not confer to the definition of ROC curves and we call them sensitivity ROC-like curves.

##### 2.1.1 Reference-dependent evaluation without consideration of alignment quality

Our main contribution is in the development of an approach to delineate a set of 'true', 'false' and 'undefined' pairs. We aimed to construct a scoring function that would provide a high-quality discrimination between structurally similar and dissimilar domains. To minimize the number of 'undefined' pairs we probed a number of sequence and structure scores and used Support Vector Machine (SVM) [46] to select the best of those scores (= features) and to optimize their weights in a linear combination.

###### Sequence and structure scores

For each domain pair, we compute structure superpositions with three programs (DALI, FAST, and TM) and use these superpositions to derive structure- and sequence-based similarity scores. Ten types of similarity scores were computed for each pair on all three structure alignments. Sequence scores were two identity measures (*pid*(1) and *pid*(2), see above), BLOSUM score, and alignment coverage; structure scores included two modifications of GDT_TS [19] (GDT_TS normalized by the length of the shorter domain and GDT_TS normalized by the length of the aligned region), match index [47], DALI Z-score [48], TM score [39] and FAST score [27]. When used individually, none of these scores provides a satisfactory discrimination between similar and dissimilar domains. Thus to construct a better composite measure, we use SVM.

###### SVM feature selection

We seek to discriminate between clearly similar domains (the same SCOP superfamily) and clearly dissimilar domains (different SCOP classes). As a training set, we randomly select 2000 pairs of SCOP domains from four major SCOP classes (Classes a, b, c and d). Among them, 1000 pairs belong to difference SCOP classes and are labeled as negative for SVM training; 1000 pairs belong to the same SCOP superfamilies and are labeled positive. When SVM is trained in the initial setting, with the full set of 30 input features (all combinations of 10 scores described in the previous section and 3 variants of structure alignments), the resulting classification accuracy is 94.8%. We find that classification accuracy increases when some of the features are removed from the set. In order to determine the importance of each feature for the discrimination, we transform the raw similarity scores into Z-scores, train SVM and find the weight for each feature in the resulting SVM model. According to these weights, five features dominate the classification (relative contributions indicated in brackets): DALI Z-score (52%), FAST score (32%), coverage of FAST alignment (6.5%), GDT_TS of TM alignment (5.0%), and BLOSUM score of DALI alignment (4.6%). Combination of these five features produces the best SVM prediction accuracy of 95.7%.

###### Selection of SVM score boundaries for the 'undefined' category

The resulting SVM makes a binary classification of domain pairs into the categories of similar and dissimilar. However, there is a number of domain pairs that share short regions of similarity but are poor global structural templates for each other (for example, Rossmann-type folds *vs*. TIM barrels). Forcing such cases to either of the two categories might bias the evaluation protocol. Therefore, following others [7,29], we use the third category of 'undefined' relations and establish the corresponding lower and higher thresholds of SVM score to define the three areas: dissimilar, unknown and similar.

To determine these thresholds, we select four pairs of SCOP folds (one per major SCOP class) that share some local similarity but are globally different (Table 2 and 3). To define the higher threshold, we consider the distribution of SVM scores between folds. All four distributions are located near the original SVM cutoff for the binary classification (not shown). We aim to classify these relationships as dissimilar ('false') and thus use 95% percentile of corresponding score distributions as the upper boundary of the 'unknown' zone (Table 2). To define the lower threshold, we select the more diverse fold in each pair and consider the distribution of SVM scores within this fold. We aim to classify these relationships as similar ('true') and thus use 5% percentile of corresponding score distributions as the lower boundary of the 'unknown' zone (Table 3).

**Table 2:** Higher threshold of SVM scores for defining 'unknown' category of similarity relations between domains

SCOP class	SCOP fold pairs	SVM score at 95% percentile
α/β	Rossmann-like folds vs. TIM barrel	0.7
α + β	Ferredoxin-like vs. IF3-like	0.6
All α	Four-helical up-and-down bundle vs. globin-like	0.8
All β	OB-fold vs. SH3-like barrel	0.3

**Average**		**0.6**

The average value in the table is the determined higher boundary for the distributions of scores between folds that share local similarity but are not global structure templates for each other (false positives).

**Table 3:** Lower threshold of SVM scores for defining 'unknown' category of similarity relations between domains

SCOP class	SCOP fold(s)	SVM score at 5% percentile
α/β	Rossmann-like folds (77 SCOP folds)	-0.8
α + β	Ferredoxin-like	-1.0
All α	Four-helical up-and-down bundle	0.4
All β	OB-fold	-1.1

**Average**		**-0.6**

The average value in the table is the determined lower boundary for the distributions of scores between folds that share local similarity but are not global structure templates for each other (false positives).

Finally, we combine our SVM classification with the expert similarity definitions in SCOP (Fig. 3A). A domain pair is automatically considered similar if both domains belong to the same SCOP superfamily. Otherwise, the pair is classified according to the SVM score as similar (score higher than the high threshold), dissimilar (score lower than the low threshold), or unknown (score between the thresholds).

##### 2.1.2 Reference-dependent evaluation with additional requirement for alignment quality

In order to evaluate the quality of a sequence-based alignment between query and hit in terms of its usefulness for structural modeling, we compare the sequence-based alignment to the structure-based (DALI-generated) reference alignment of the pair (Fig. 3B). The 3D structures of the query and the hit are superimposed according to the equivalent residues in the sequence-based alignment. All scores are calculated from this sequence-based superposition of the pair.

GDT_TS [19] and LiveBench 3dscore have been traditionally used in CASP and other assessments [21,49] to evaluate the quality of a sequence alignment. However, since these scores have only been used to rank different structure models relative to each other, no absolute cutoff has been reported to indicate a good alignment. The number of correct matches has also been used to assess alignment quality [35]. Number of correct matches is the number of residue pairs that are aligned the same way in sequence alignment as in the reference structure alignment. In order to find a reasonable cutoff for the alignment quality, we explored GDT_TS, 3dscore and the number of correct matches. Since GDT_TS and 3dscore performed similarly (data not shown), only GDT_TS and number of correct matches were used.

**Figure 7:**
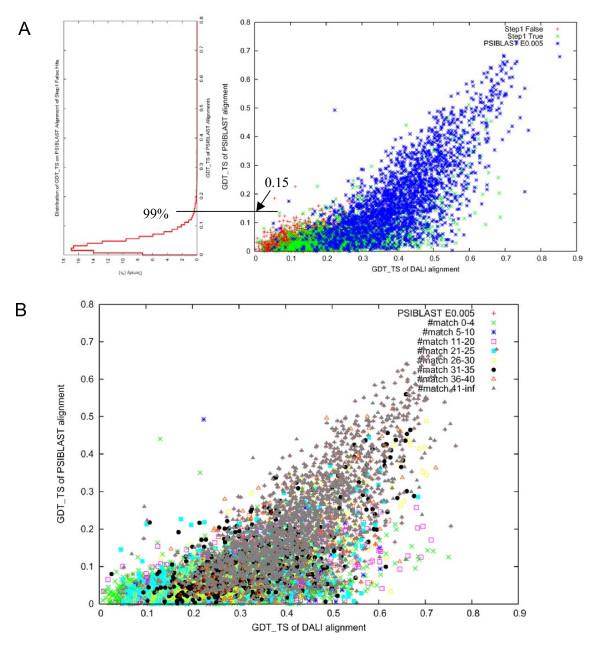
**Selection of cutoffs for alignment quality**. A. GDT_TS scores of PSI-BLAST alignment vs. GDT_TS scores of structure-based DALI alignment of the step1 false domain pairs (red), step1 true domain pairs (green) and domain pairs whose similarity can be detected by PSI-BLAST (E-value < 0.005) (blue). B. Coverage of the domain pairs with PSI-BLAST E-value < 0.005, by different ranges of number of correct matches.

All-against-all pairwise sequence alignments are generated using PSI-BLAST for 500 randomly chosen domains. PSI-BLAST is first used to find and align a family of homologs for each domain sequence (NCBI nr database, inclusion E-value cutoff of 10^-4 ^for up to 2 iterations). Then PSI-BLAST is used to compare the resulting alignments for each domain to all individual sequences from families of homologs for other domains. The sequence with the best E-value is chosen in each family. This best E-value and the corresponding sequence alignment are assigned to the comparison of the two domains. The reference-dependent template quality evaluation is applied to these alignments and the 'false' pairs are used as negative examples. PSI-BLAST alignments of all pairs with significant E-values (E-value cutoff 0.005, up to 3 iterations) are used as positive examples. Fig. 7A shows GDT_TS of structure alignment vs. sequence alignment plotted for both negative and positive examples. Since there is no clear separation between GDT_TS for negatives and positives, the alignment quality criterion consists of two components. First, the 99 percentile of GDT_TS of the negative examples, which corresponds to GDT_TS 0.15, is selected as the GDT_TS cutoff to select alignments useful for structure modeling. However, there are PSI-BLAST alignments with significant E-values having GDT_TS less than this cutoff of 0.15. This usually happens to extended alignments that include a conserved sequence motif, but with poor structural conservation outside the motif. In order not to overlook such potentially useful alignments, additional criterion of the number of correct matches is used. Alignments of the 'true' hits from the template quality evaluation are compared with structure alignments (DALI), and the number of correct matches is calculated for each alignment. We find that the alignments with the number of correct matches more than 5 cover about 97% of all PSI-BLAST alignments with significant E-values (Fig. 7B). Thus the cutoff for number of correct matches is chosen to be 5, which is consistent with a previous study [35].

Overall, if a sequence alignment has a GDT_TS higher than 0.15 or has a number of correct matches more than 5, it is considered to be useful for modeling (= true positive). Otherwise, it is considered to be 'false' (Fig. 3B).

#### 2.2 Reference-independent evaluation

We define that reference-independent evaluation does not use any other information except the alignment generated by a sequence method under evaluation and 3D structures of the aligned proteins. Thus we directly evaluate the usefulness of the sequence-based alignment for structure modeling. This evaluation is more in-line with model-to-structure comparison paradigm that bypasses the reference (be it classification database or alignment), as established by CASP experiments. However, reference-independent evaluation, despite its conceptual simplicity, is still rarely used in the field of sequence analysis (be it search or alignment methods).

We start from a pairwise sequence alignment being tested, and superimpose 3D structures of the aligned proteins to minimize RMSD for the residue equivalencies given by the sequence alignment (Fig. 3C,D). If the sequence alignment is structurally meaningful (as defined in the further subsections), the resulting superposition guided by this alignment would result in a reasonable structure similarity. In our method, we use GDT_TS score of this superposition to measure structural similarity, and evaluate usefulness of an alignment for 1) making an overall fold model (global, Fig. 3C) or 2) finding possibly short, but structurally similar fragments (local, Fig. 3D). It is important to emphasize the distinction between global/local alignments and global/local evaluation. Local alignment is defined as an alignment of two sequence fragments, versus global alignment of complete sequences. We say that evaluation is 'local' if we evaluate the quality of generated alignment (global or local) within alignment boundaries and are not concerned with alignment coverage. We say that evaluation is 'global' if we evaluate the usefulness of an alignment for modeling of the entire domain, thus we are concerned about the regions potentially not covered by a local alignment.

##### 2.2.1 Reference-independent global mode evaluation

In a global mode (Fig. 3C), we evaluate the alignment quality over the entire length of the query domains. The GDT_TS cutoff (0.15) from the reference-dependent evaluation may provide a reasonable cutoff for the global structural template quality because this value provides exclusion of 99% of the hits that do not share fold similarity with the query (Fig. 7A). Since the reference-independent evaluation is based on sequence alignment only, we cannot use the number of correct matches according to a reference. GDT_TS score alone is probably a more stringent criterion than the reference-dependent evaluation, which is a union of GDT_TS and the number of correct matches. However, with reference-independent evaluation we do not use SCOP database or SVM score to define a set of reasonable templates, and thus some alignments between proteins with different folds may be considered 'true' with the GDT_TS cutoff of 0.15. These will correspond to partial, but close, structural matches.

##### 2.2.2 Reference-independent local mode evaluation

Reference-dependent evaluation is necessarily done in a global mode, because it relies on a single structure alignment generated by DALI. However, there could be many reasonable local structure alignments for a pair of proteins, i.e. each helix can be superimposed with another helix. Local evaluation can be easily done in a reference-independent way (Fig. 3D).

We define a local version of GDT_TS score as:

lGDT_TS=1Lali∑Lali(p1+p2+p4+p84),
 MathType@MTEF@5@5@+=feaafiart1ev1aaatCvAUfKttLearuWrP9MDH5MBPbIqV92AaeXatLxBI9gBaebbnrfifHhDYfgasaacH8akY=wiFfYdH8Gipec8Eeeu0xXdbba9frFj0=OqFfea0dXdd9vqai=hGuQ8kuc9pgc9s8qqaq=dirpe0xb9q8qiLsFr0=vr0=vr0dc8meaabaqaciaacaGaaeqabaqabeGadaaakeaacqWGSbaBcqWGhbWrcqWGebarcqWGubavcqGGFbWxcqWGubavcqWGtbWucqGH9aqpdaWcaaqaaiabigdaXaqaaiabdYeamnaaBaaaleaacqWGHbqycqWGSbaBcqWGPbqAaeqaaaaakmaaqafabaWaaeWaaeaadaWcaaqaaiabdchaWnaaBaaaleaacqaIXaqmaeqaaOGaey4kaSIaemiCaa3aaSbaaSqaaiabikdaYaqabaGccqGHRaWkcqWGWbaCdaWgaaWcbaGaeGinaqdabeaakiabgUcaRiabdchaWnaaBaaaleaacqaI4aaoaeqaaaGcbaGaeGinaqdaaaGaayjkaiaawMcaaaWcbaGaemitaW0aaSbaaWqaaiabdggaHjabdYgaSjabdMgaPbqabaaaleqaniabggHiLdGccqGGSaalaaa@5464@

where *p*_1_, *p*_2_, *p*_4_, *p*_8 _are the fractions, normalized by the query length, of aligned Cα atoms that are within the distance of 1, 2, 4, 8Å from each other in an RMSD-minimized superposition, and *L*_*ali *_is the length (number of residues) of the aligned region. However, we cannot use lGDT_TS directly because it has a significant dependency on aligned domain length. If the aligned region is very short, essentially all residues in the aligned region will be very close to each other and the resulting lGDT_TS will be artificially large. In the extreme case, if there is only one residue aligned, the lGDT_TS will be a perfect score of 1.0. This length dependency effect is also present in the global GDT_TS, but it does not affect the global mode evaluation much because the length of the query, and not of an alignment) is used in normalization. When the aligned region is very short compared to the length of the query, the resulting global GDT_TS is small. Thus the global GDT_TS favors long alignments, while the local GDT_TS favors short alignments.

**Figure 8:**
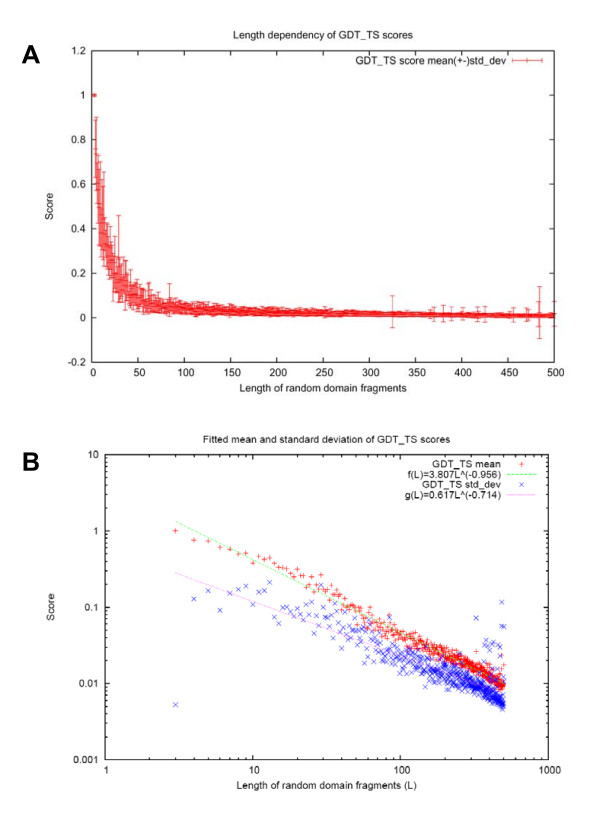
**Dependency of GDT_TS score on the alignment length**. A: Average GDT_TS scores plotted for different bins of length of randomly aligned domains. B: Approximations of mean and standard deviation of GDT_TS scores as functions of length.

In order to correct for this length effect in lGDT_TS, we study the length-dependency of lGDT_TS. For a particular length L, we randomly select 1000 pairs of domain fragments of the length L from our testing dataset. Each pair is aligned to equivalence residues in their numerical order (first residue to the first residue, second to the second, etc), and minimum RMSD structure superposition was performed based on this alignment. lGDT_TS score is calculated based on this superposition. The study of lengths from 3 to 500 reveals power-law dependency of the mean and the standard deviation of lGDT_TS on length. (Fig. 8). Using the function *f(L) = cL*^*b *^to fit the lGDT_TS mean and sd (Fig. 8B), we obtain the following formulas

*mean *_1GDT_TS(*L*) = 3.807*L*^-0.956^, sd_1GDT_TS(*L*) = 0.617*L*^-0.714^

The raw lGDT_TS score of sequence alignment of length *L *is transformed to a length-corrected Z-score. By using this Z-score of lGDT_TS, we are able to find alignments with lGDT_TS scores significantly higher than random for that length. Alignments with Z-scores higher than 3 are considered to be of reasonable quality (Fig. 8B) and are labeled 'true' in local evaluation (Fig. 3D).

### 3. Evaluation of alignment quality

To evaluate the quality of sequence alignments, we consider all structurally similar domain pairs and bin them by sequence identity in DALI structural alignments. In each identity bin, we calculate and compare several reference-dependent measures of alignment quality proposed earlier [3,8,50], using DALI alignments as reference, as well reference-independent measures that we derive from GDT_TS score (see above). In both reference-dependent and reference-independent protocols, the assessment is made from global and from local perspectives.

#### 3.1 Reference-dependent evaluation of alignment quality

The quality from the modeler's point of view (*Q*_modeller_[50]) is the ratio of the number of correctly aligned positions to the total number of positions in the evaluated alignment. The quality from the developer's point of view (*Q*_developer_[50]) is the ratio of the number of correctly aligned positions to the number of positions in the structural alignment. For local alignments, it is reasonable to assess the local prediction for only those regions of the structural alignment that are included in the evaluated alignment. Thus in addition to Q_developer_, we use a measure of 'local accuracy' [35]: Q_local _= *N*_*acc*_/*L*, the ratio of the number of correctly aligned positions *N*_*acc *_to the length *L *of the region in the structural alignment that includes the pairs of residues from the alignment under evaluation. Q_local _is close to 1.0 for alignments with the correct prediction of structural matches, even if they are very short. To assess directly the length of the region covered by the alignment, we introduce an additional measure of coverage independent of accuracy. To calculate the coverage, we determine the length of the region in the structural alignment that includes all residues from the evaluated alignment and divide it by the overall length of the structural alignment.

#### 3.2 Reference-independent evaluation of alignment quality

Based on evaluated alignments and domain 3D structures, we calculate original GDT_TS score (normalized by full domain length) and 'local' GDT_TS score normalized by the alignment length and adjusted according to the length dependency of random GDT_TS scores (see section 2.2.2).

## Authors' contributions

YQ carried out the methodology development and ROC-based evaluation of methods for sequence comparison, as well as drafted the manuscript. RIS participated in the methodology design, evaluation, and writing the manuscript. YW carried out the evaluation of homology detection using paired tests, and of alignment quality. BHK provided the structure-based sequence alignments and scores. NVG carried out the methodology design and participated in drafting the manuscript. All authors read and approved the final manuscript.

## Supplementary Material

Additional file 1 domain_seqs.fa. Sequences of protein domains included in the benchmark set. Click here for file
